# Association between the 2012 Health and Social Care Act and specialist visits and hospitalisations in England: A controlled interrupted time series analysis

**DOI:** 10.1371/journal.pmed.1002427

**Published:** 2017-11-14

**Authors:** James A. Lopez Bernal, Christine Y. Lu, Antonio Gasparrini, Steven Cummins, J. Frank Wharham, Steven B. Soumerai

**Affiliations:** 1 Department of Social and Environmental Health Research, London School of Hygiene and Tropical Medicine, London, United Kingdom; 2 Department of Population Medicine, Harvard Medical School and Harvard Pilgrim Health Care Institute, Boston, Massachusetts, United States of America; Edinburgh University, UNITED KINGDOM

## Abstract

**Background:**

The 2012 Health and Social Care Act (HSCA) in England led to among the largest healthcare reforms in the history of the National Health Service (NHS). It gave control of £67 billion of the NHS budget for secondary care to general practitioner (GP) led Clinical Commissioning Groups (CCGs). An expected outcome was that patient care would shift away from expensive hospital and specialist settings, towards less expensive community-based models. However, there is little evidence for the effectiveness of this approach. In this study, we aimed to assess the association between the NHS reforms and hospital admissions and outpatient specialist visits.

**Methods and findings:**

We conducted a controlled interrupted time series analysis to examine rates of outpatient specialist visits and inpatient hospitalisations before and after the implementation of the HSCA. We used national routine hospital administrative data (Hospital Episode Statistics) on all NHS outpatient specialist visits and inpatient hospital admissions in England between 2007 and 2015 (with a mean of 26.8 million new outpatient visits and 14.9 million inpatient admissions per year). As a control series, we used equivalent data on hospital attendances in Scotland. Primary outcomes were: total, elective, and emergency hospitalisations, and total and GP-referred specialist visits. Both countries had stable trends in all outcomes at baseline. In England, after the policy, there was a 1.1% (95% CI 0.7%–1.5%; *p* < 0.001) increase in total specialist visits per quarter and a 1.6% increase in GP-referred specialist visits (95% CI 1.2%–2.0%; *p* < 0.001) per quarter, equivalent to 12.7% (647,000 over the 5,105,000 expected) and 19.1% (507,000 over the 2,658,000 expected) more visits per quarter by the end of 2015, respectively. In Scotland, there was no change in specialist visits. Neither country experienced a change in trends in hospitalisations: change in slope for total, elective, and emergency hospitalisations were −0.2% (95% CI −0.6%–0.2%; *p* = 0.257), −0.2% (95% CI −0.6%–0.1%; *p* = 0.235), and 0.0% (95% CI −0.5%–0.4%; *p* = 0.866) per quarter in England. We are unable to exclude confounding due to other events occurring around the time of the policy. However, we limited the likelihood of such confounding by including relevant control series, in which no changes were seen.

**Conclusions:**

Our findings suggest that giving control of healthcare budgets to GP-led CCGs was not associated with a reduction in overall hospitalisations and was associated with an increase in specialist visits.

## Abstract 2

Using a controlled interrupted time series analysis, James Lopez Bernal and colleagues investigate the changes in specialist visits and hospitalizations following the healthcare reforms of the 2012 Health and Social Care Act in England.

## Author summary

**Why was this study done?:**

- In 2012, the government introduced major reforms to the National Health Service (NHS) in England, which handed budgets for specialist care to GP-led organisations known as Clinical Commissioning Groups.
- This gave GPs a major new role in purchasing hospital-based specialist medical care for patients, in addition to their existing role as “gatekeepers” to specialist care.
- An expected effect of this policy was that there would be a shift in care away from expensive hospital-based care and towards the community.
- Our study aimed to evaluate the potential impact of these reforms on levels of hospital activity including outpatient visits to specialists and inpatient admissions.

**What did the researchers do and find?:**

- We examined trends in all NHS specialist visits and hospital admissions between 2007 and 2015 in order to examine changes in trends following the reforms.
- We included equivalent trends in Scotland, where the reforms did not occur, as a control series.
- We found no change in hospital admissions in either country.
- However, in England we found an increase in the trend of outpatient specialist visits following the reforms, equivalent to approximately 3.7 million additional specialist visits between the time the policy was implemented and the end of the study period (compared to expected).

**What do these findings mean?:**

- Our findings suggest that giving control of healthcare budgets to GP-led CCGs was not linked to a decrease in hospital admissions and was associated with an increase in outpatient specialist visits.
- Further research is needed to establish the appropriateness of these visits and the reasons for the increase.
- However, these findings suggest that other interventions may be needed in order to shift more patient care into the community.

## Introduction

The 2012 Health and Social Care Act (HSCA) in England has been described as “the biggest and most far reaching [reorganisation] in the history of the NHS” [1, 2]. The reforms centred around the introduction of general practitioner (GP) led Clinical Commissioning Groups (CCGs), which received about two-thirds of the National Health Service (NHS) budget (£66.8 billion in 2015–2016) to commission (plan and contract) secondary care, including hospital and specialist services [1]. CCGs represent all GP practices in their local area, and the key difference from the previous commissioning structures was purported to be a major new role for GPs as key decision makers in the commissioning process [1, 3, 4].

Health policy experts and parliamentary and professional bodies have hypothesised that GP-led commissioning could potentially lead to reductions in referrals to specialist care, as either an intended or unintended consequence of the Act [5–10]. They theorise that by giving the gatekeepers, who control access to specialist care, a greater role in budget holding and the purchasing of specialist care, they may be incentivised to reduce referrals [5, 6]. Indeed, Smith and Mays suggest that the primary rationale for GP-led commissioning is to encourage a shift away from expensive secondary care towards more community-based care [6]. Furthermore, 2 out of the 3 main reasons cited by the government for introducing the reforms centred around a need to control costs, although the mechanisms by which GP-led CCGs would achieve cost savings were not made explicit [4]. While the potential for much-needed cost savings in the NHS as a result of reduced secondary care activity has been viewed positively, some—including the National Audit Office and the Royal College of Surgeons—have raised concerns that a reduction in referrals as a consequence of the HSCA and policies introduced by CCGs could result in inequitable rationing of care and missed diagnoses and that their role in commissioning presents GPs with a conflict of interest [7–10].

CCGs and GPs could reduce secondary care activity through various means, including restricting referral criteria, developing community-based care models, investing in preventative healthcare, or promoting services to prevent readmissions [6, 9, 10]. There also exist potential incentives for them to do so: reducing expensive care would allow CCGs to invest savings in other services. Furthermore CCGs are required to maintain a surplus; otherwise, they cannot access additional funding in the form of a “Quality Premium” of up to £5 per person within the population covered by the CCG person [11]. In addition, there are incentives to individual GPs: savings from reduced specialist visits and hospitalisations could allow investment in community-based services provided by GP practices themselves; also, some CCGs have introduced direct payments of £6,000–£11,000 to GP practices for reducing referral rates [7, 8]. Nevertheless, whether these provide a real incentive in practice depends on how engaged GPs feel with the new commissioning organisations, how much responsibility they feel for their budgets, and how much influence they have on the commissioning process. Previous policies that have begun with an intention to place GPs at the centre of commissioning have ultimately resulted in the formation of bureaucratic bodies that have become detached from local practitioners [6]. Furthermore, given existing evidence that increasing GP workload may increase referral rates, it is possible that the increased administrative burden associated with their new commissioning role could instead result in an increase in referrals [12, 13].

We use a controlled interrupted time series (CITS) design to compare changes in the trends of specialist referrals and hospital admissions in England before and after the HSCA with those in Scotland, where the reforms did not occur. We hypothesise that the 2012 HSCA was associated with a reduction in specialist visits and hospitalisations.

## Methods

### Ethics

Ethical approval was obtained from the London School of Hygiene and Tropical Medicine Observational/Interventions Research Ethics Committee (LSHTM Ethics Ref: 10505).

### The intervention

The 2012 HSCA introduced broad ranging and complex reforms to the NHS and public health services in England. These have been described in more detail elsewhere [1, 2, 4, 14, 15]. The principal change was in the way secondary care services were commissioned within the NHS. Prior to 2012, regional healthcare administrative bodies known as primary care trusts (PCTs) and Strategic Health Authorities (SHAs) were responsible for all commissioning. These were abolished as part of the Act and were replaced by CCGs. CCGs are led by a governing body, which includes a representative from each member GP practice, lay members, a secondary care doctor, and a registered nurse [3]. CCGs were first introduced in shadow form (working alongside PCTs) in April 2012 following the enactment of the HSCA; they then took over full budgetary responsibility in March 2013 [1].

### Control

While a control is not required in interrupted time series studies, the primary comparison being between preintervention and postintervention trends within the study population, a control population can help to exclude additional confounding events and cointerventions. Healthcare is a largely devolved power in the United Kingdom and the HSCA only applied to England; therefore, we considered the other 3 nations of the UK (Northern Ireland, Scotland, and Wales) as potential controls. These are neighbouring countries with similar population demographics (S1 Table), similar health systems, and shared political structures. Data equivalent to those in England were not available from Northern Ireland; therefore, it was excluded. We chose Scotland as the control, as preintervention data were more stable than for Wales. We also include an analysis as a supplementary appendix with Wales as the control (S2 Table, S1 and S2 Figs).

### Data and study population

We obtained quarterly data on all hospital admissions and outpatient specialist visits in NHS hospitals in England between April 2007 and December 2015 from the Health and Social Care Information Centre: Hospital Episode Statistics (HES) [16]. Hospital admissions include all inpatients in NHS hospitals as well as NHS-funded inpatients in the private sector. NHS outpatient activity in England is hospital based; specialist visit data include outpatients in English NHS hospitals and NHS-funded outpatients in the private sector. Our outcomes were total hospital admissions, elective (planned) and emergency (unplanned) admissions, total first specialist visits (excluding follow-up appointments), and GP-referred first specialist visits. Equivalent data for Scotland were obtained from the NHS Scotland Information Services Division: Scottish Medical Records (SMR) [17]. We obtained demographic data for England and Scotland from the Office for National Statistics including midyear population estimates (for denominators), age and sex distribution, crude birth rate, and crude death rate [18]. The Scottish hospital admission data did not include obstetric and psychiatric hospitals and the outpatient visit data did not include visits to nurses, dentists, or other allied health professionals. We therefore excluded these categories from the English data to make the 2 datasets comparable. Data quality reports identified a coding error in the outpatient data prior to April 2010 (3 years before the introduction of the policy); we therefore excluded these data from the analysis [19]. The raw data are provided in the supplementary appendix (S1 and S2 Data). A complete list of the data codes and algorithms used in the data extraction is also provided in the supplementary appendix (S3 and S4 data).

### Statistical analysis

We used a CITS design, which allowed us to control both for preintervention trends in the outcome and for potential confounding events that would have affected both the control and the study groups. Although a Poisson distribution is assumed for individual counts, we had very large numbers and the aggregate data were well approximated by a Gaussian distribution (log transformed). Therefore, we used a simple linear segmented regression model to estimate the change in trend in hospital admissions and outpatient visits following the introduction of the HSCA [20]. In order to account for the year during which CCGs were in shadow form, we allowed a one-year phase-in period by excluding the second quarter of 2012 to the first quarter of 2013 from the analysis. We modelled the association as a slope change rather than an immediate level change because choice of providers and referral patterns were likely to change gradually when existing contracts expired and new models of care developed [1]. Adjustments were made for any seasonal effect using a Fourier term [20].

We first estimated the slope changes in England and in Scotland independently. We then used an interaction model for the CITS to estimate the additional trend change in England over and above any change in Scotland, while controlling for any difference in the preintervention trends of the 2 groups (S1 Text). We examined the preintervention data a priori for linearity and autocorrelation at different lags using scatterplots, plots of residuals, and partial autocorrelation functions [20]. A linear trend provided a reasonable fit for all outcomes in the primary model. We included an autoregressive term at the appropriate lag to adjust for any detected autocorrelation. All analyses were conducted using Stata version 14.

### Protocol

The original study protocol from the ethics application is available as a supplementary appendix (S1 Protocol). The analysis has only differed from this protocol in that Scotland was selected as the primary control, as it had the most stable data; Wales was included as an additional control following reviewers’ recommendations. Northern Ireland was not included as equivalent data to that in England was not available. Furthermore, in this protocol, we also proposed including patient experience measures as secondary outcomes; this was ultimately not included within the current study but we plan to conduct a future study looking at the potential impact on patient experience.

### Reporting

This study is reported as per the REporting of studies Conducted using Observational Routinely-collected health Data (RECORD) Statement (S1 Checklist).

## Results

### Population characteristics

Age and sex distributions were similar in both England and Scotland (Table 1 and S1 Table). Both populations were slowly aging; the proportion aged 60 or older increased from 21.6% to 23.0% in England and from 22.3% to 24.0% in Scotland. The crude birth rate was consistently about 1.8 per 1,000 higher in England than in Scotland while the crude death rate was consistently about 1.5 per 1,000 lower.

**Table 1:** Population characteristics of England and Scotland, 2007–2014.

		England			Scotland		
		2007	2011	2014	2007	2011	2014
	*N*	51,381,100	53,107,200	54,316,600	5,170,000	5,299,900	5,347,600
Age (%)							
	0–19	24.2	23.9	23.8	23.0	22.3	21.7
	20–39	27.3	26.9	26.6	26.3	25.9	25.9
	40–59	26.8	26.7	26.8	28.5	28.6	28.4
	60–79	17.1	17.8	18.2	18.1	18.9	19.4
	80+	4.5	4.6	4.8	4.2	4.4	4.6
Sex (%)							
	Males	49.2	49.2	49.3	48.3	48.5	48.6
	Females	50.8	50.8	50.7	51.7	51.5	51.4
Crude birth rate (per 1,000)		12.8	13.0	12.2	11.2	11.1	10.6
Crude death rate (per 1,000)		9.2	8.5	8.6	10.8	10.1	10.1

### Changes in outpatient specialist visits

**Fig 1:**
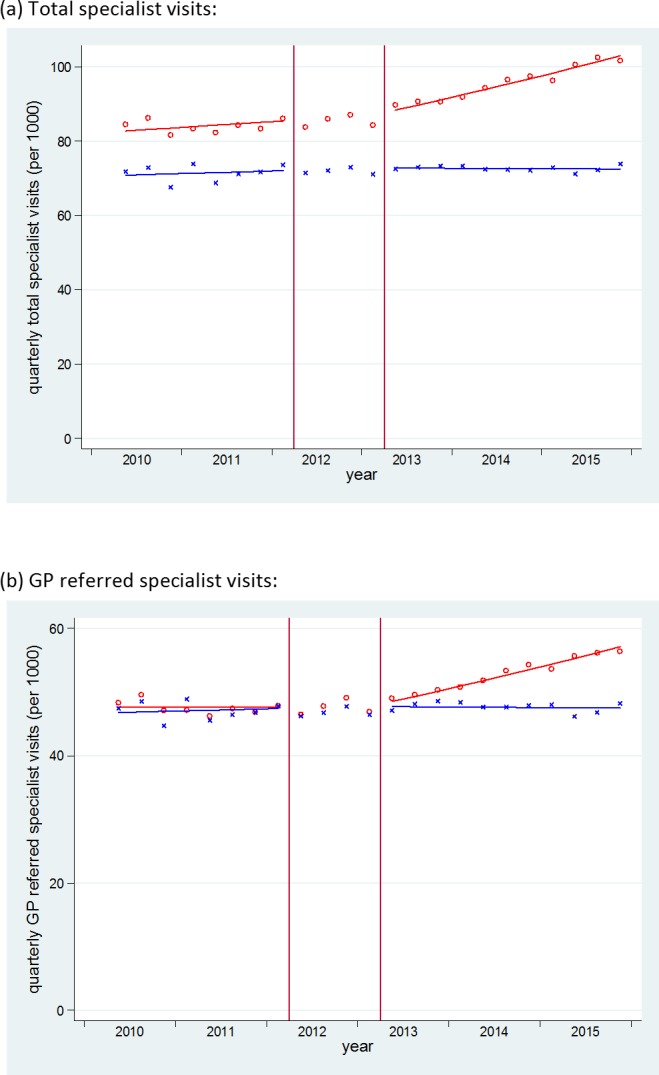
Time series of outpatient specialist visits in England and Scotland. Red o = England, blue x = Scotland. Lines = deseasonalized linear trend. Vertical lines delineate the intervention phase (between quarter 2 [Q2] 2012 and Q2 2013). The data underlying this figure are presented in Table 3. GP, general practitioner.

Changes in trends of specialist visits are shown in Fig 1 and Table 2. Absolute counts and the rate per 1,000 for each quarter are presented in Table 3. In England, total specialist visits rose slowly by 0.5% per quarter (from 84.7 per 1,000 in quarter 2 [Q2] 2010 to 87.2 per 1,000 in Q1 2012) in the baseline. After the intervention, they rose approximately 3.6 times faster at 1.5% per quarter (from 90.0 per 1,000 in Q2 2013 to 104.6 per 1,000 in Q4 2015). This was equivalent to an increase in slope (additional quarterly increase) of 1.1% (95% CI 0.7%–1.5%), which resulted in a 12.7% higher rate of specialist visits (647,000 additional visits) by the end of the postintervention period in Q4 2015, compared to the underlying (counterfactual) trend. The slope increase was even more marked for GP-referred visits. During the preintervention period, these had a flat trend at 48.3 visits per 1,000 per quarter (trend 1.000, 95% CI 0.998–1.002). After the intervention, this trend increased by 1.6% per quarter (from 49.1 per 1,000 in Q2 2013 to 57.6 per 1,000 in Q4 2015). This was equivalent to an increase in slope of 1.6% (95% CI 1.2%–2.0%) per quarter, which resulted in a 19.1% higher than expected rate of specialist visits (507,000 additional visits) by the end of the study period. For those outcomes that showed strong evidence of a trend change (total and GP-referred specialist visits in England), we have presented observed compared to expected counts in the postintervention period in Table 4. Total specialist visits had weak evidence of seasonal effect with peaks during Q3 (Fourier sin wave *p* = 0.055, cos wave *p* = 0.010).

**Table 2:** Changes in trend in specialist visits and hospitalisations following the intervention.

		Trend change England			Trend change Scotland			Trend change England versus Scotland		
		Effect	95% CI	*p*-value	Effect	95% CI	*p*-value	Effect	95% CI	*p*-value
Outpatient specialist visits										
	Total	**1.011**	**[1.007,1.015]**	**<0.001**	0.997	[0.991,1.003]	0.390	**1.014**	**[1.006,1.021]**	**<0.001**
	GP referred	**1.016**	**[1.012,1.020]**	**<0.001**	0.998	[0.991,1.004]	0.491	**1.018**	**[1.010,1.027]**	**<0.001**
Inpatient hospitalizations										
	Total	0.998	[0.994,1.002]	0.257	0.998	[0.993,1.002]	0.294	1.000	[0.994,1.006]	0.980
	Elective	0.998	[0.994,1.001]	0.235	0.999	[0.993,1.004]	0.625	0.999	[0.992,1.005]	0.670
	Emergency	1.000	[0.995,1.004]	0.866	0.995	[0.989,1.001]	0.114	1.002	[0.995,1.010]	0.497

Coefficients for trend change are relative change in the slope gradient following the intervention. Trend change study versus control is the slope change in England over and above any change in Scotland accounting for differences in baseline trends. All segmented regression models used log transformed Gaussian distribution and *p*-values were derived from *z*-tests. Cells in bold indicate strong evidence of an effect (p<0.05).

**Abbreviation:** GP = general practitioner.

**Table 3:** Absolute counts and rates of specialist visits in each quarter.

		England				Scotland			
		Total		GP referred		Total		GP referred	
Year	Quarter	Count (x10^5)	Rate (per 1,000)	Count (x10^5)	Rate (per 1,000)	Count (x10^5)	Rate (per 1,000)	Count (x10^5)	Rate (per 1,000)
2010	2	44.55	84.7	25.42	48.3	3.73	70.9	2.46	46.8
2010	3	44.84	85.0	25.48	48.3	3.74	71.0	2.47	46.9
2010	4	45.13	85.4	25.54	48.3	3.76	71.2	2.48	47.0
2011	1	45.41	85.8	25.59	48.3	3.77	71.4	2.49	47.1
2011	2	45.71	86.1	25.65	48.3	3.79	71.5	2.50	47.1
2011	3	45.99	86.5	25.70	48.3	3.80	71.7	2.50	47.2
2011	4	46.26	86.9	25.75	48.3	3.81	71.9	2.51	47.3
2012	1	46.54	87.2	25.80	48.3	3.83	72.1	2.52	47.4
2012	2								
2012	3								
2012	4								
2013	1								
2013	2	48.47	90.0	26.44	49.1	3.87	72.7	2.54	47.7
2013	3	49.30	91.4	26.92	49.9	3.88	72.7	2.54	47.7
2013	4	50.15	92.8	27.41	50.7	3.88	72.7	2.54	47.7
2014	1	51.02	94.2	27.91	51.5	3.88	72.7	2.54	47.6
2014	2	51.90	95.6	28.41	52.3	3.88	72.7	2.55	47.6
2014	3	52.80	97.1	28.93	53.2	3.89	72.6	2.55	47.6
2014	4	53.71	98.5	29.46	54.0	3.89	72.6	2.55	47.6
2015	1	54.64	100.0	29.99	54.9	3.89	72.6	2.55	47.5
2015	2	55.58	101.5	30.54	55.8	3.89	72.6	2.55	47.5
2015	3	56.54	103.1	31.09	56.7	3.89	72.6	2.55	47.5
2015	4	57.51	104.6	31.65	57.6	3.90	72.5	2.55	47.5

**Abbreviation:** GP, general practitioner.

Modelled counts and trends after adjusting for seasonality and autocorrelation.

**Table 4:** Observed and expected counts for specialist visits in the postintervention period in England.

		Total visits (x10^5)				GP referred visits (x10^5)			
Year	Quarter	Expected	Observed	Difference	Percent difference	Expected	Observed	Difference	Percent difference
2013	2	47.95	48.47	0.52	1.1%	26.03	26.44	0.42	1.6%
2013	3	48.25	49.30	1.06	2.2%	26.08	26.92	0.84	3.2%
2013	4	48.55	50.15	1.60	3.3%	26.13	27.41	1.27	4.9%
2014	1	48.85	51.02	2.17	4.4%	26.19	27.91	1.72	6.6%
2014	2	49.16	51.90	2.74	5.6%	26.24	28.41	2.17	8.3%
2014	3	49.47	52.80	3.32	6.7%	26.30	28.93	2.63	10.0%
2014	4	49.78	53.71	3.93	7.9%	26.36	29.46	3.10	11.8%
2015	1	50.10	54.64	4.54	9.1%	26.41	29.99	3.58	13.5%
2015	2	50.42	55.58	5.17	10.2%	26.47	30.54	4.07	15.4%
2015	3	50.73	56.54	5.81	11.5%	26.53	31.09	4.56	17.2%
2015	4	51.05	57.51	6.47	12.7%	26.58	31.65	5.07	19.1%

**Abbreviation:** GP, general practitioner.

Expected counts are those expected if there had been no trend change from the pre- to postintervention period (i.e., the counterfactual). Observed counts are those modelled after adjusting for seasonality and autocorrelation. Difference is the additional number of visits in a given quarter over those expected. Percent difference is the additional number of visits expressed as a percentage of the expected number of visits.

Specialist visits in Scotland, however, showed no significant change after the policy. Total specialist visits and GP-referred specialist visits almost level at about 72 per 1,000 per quarter (preintervention trend 1.002, 95% CI 0.999–1.006; postintervention trend 1.000, 95% CI 0.996–1.003) and 47 per 1,000 per quarter (preintervention trend 1.002, 95% CI 0.998–1.005, postintervention trend 1.000, 95% CI 0.996–1.003), respectively, throughout the study period.

After controlling for trends in Scotland, the CITS analysis produced similar results. The magnitude of the change in slope in total specialist visits in England increased slightly to 1.4% (95% CI 0.6%–2.1%) per quarter (a 15.9% higher rate by the end of the study period). The change in trend in GP-referred specialist visits increased to 1.9% (95% CI 1.1%–2.7%) per quarter (a 22.5% higher rate than expected by the end of the study period).

The magnitude of the differential increase in trend in England was even greater when using Wales as a control series, although this was partly due to an independent reduction in the trend in Wales (S2 Table and S1 Fig).

### Changes in inpatient hospitalisations

**Fig 2:**
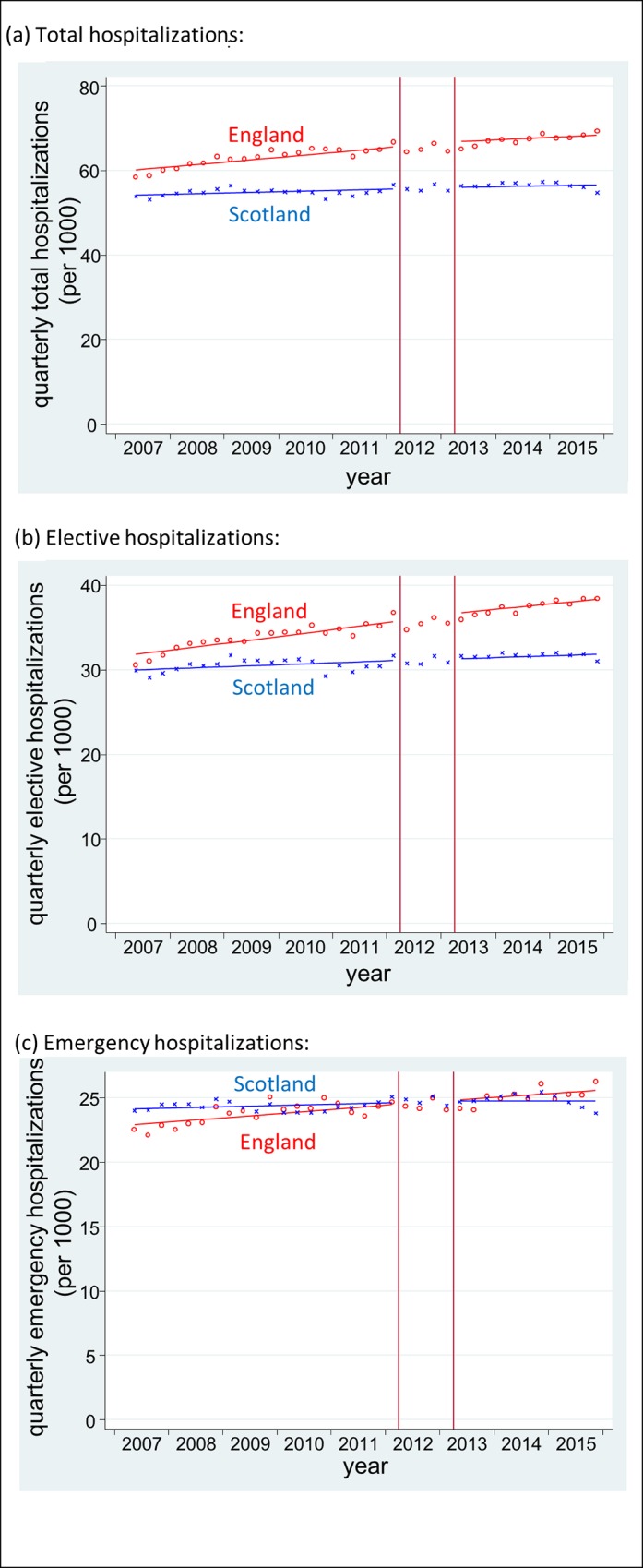
Time series of inpatient hospitalisations in England and Scotland. Red o = England, blue x = Scotland. Lines = deseasonalized linear trend. Vertical lines delineate the intervention phase (between quarter 2 [Q2] 2012 and Q2 2013). The data underlying this figure are presented in Table 5.

Changes in trends in hospitalisations following the HSCA are shown in Fig 2 and Table 2. Absolute counts and the rate per 1,000 for each quarter are presented in Table 5. In England, there were slowly increasing trends in all hospitalisations during the baseline period. Total hospitalisations increased by 0.5% per quarter (from 60.1 per 1,000 in Q2 2007 to 65.5 per 1,000 in Q1 2012), elective admissions increased by 0.6% per quarter (from 31.7 to 35.7 per 1,000), and emergency admissions increased by 0.3% per quarter (from 22.9 to 24.5 per 1,000). Total hospitalisations and emergency hospitalisations had a seasonal effect with winter peaks in Q4 (emergency hospitalisations Fourier terms: sin wave *p* = 0.002, cos wave *p* = 0.039). There were no statistically significant changes in any of these trends following the HSCA. Slope changes were −0.2% (95% CI −0.6%–0.2%), −0.2% (95% CI −0.6%–0.1%), and 0.0 (95% CI −0.5%–0.4%) per quarter for total, elective, and emergency hospitalisations, respectively.

**Table 5:** Absolute counts and rates of hospitalisations in each quarter.

		England						Scotland					
		Total		Elective		Emergency		Total		Elective		Emergency	
Year	Quarter	Count (x10^5)	Rate (per 1,000)	Count (x10^5)	Rate (per 1,000)	Count (x10^5)	Rate (per 1,000)	Count (x10^5)	Rate (per 1,000)	Count (x10^5)	Rate (per 1,000)	Count (x10^5)	Rate (per 1,000)
2007	2	30.87	60.1	16.29	31.7	11.77	22.9	2.79	54.0	1.55	29.9	1.24	24.1
2007	3	31.08	60.4	16.43	31.9	11.84	23.0	2.80	54.1	1.55	30.0	1.25	24.1
2007	4	31.29	60.7	16.57	32.1	11.91	23.1	2.81	54.2	1.56	30.1	1.25	24.2
2008	1	31.50	61.0	16.71	32.3	11.97	23.2	2.82	54.3	1.56	30.1	1.26	24.2
2008	2	31.71	61.2	16.84	32.5	12.04	23.2	2.83	54.4	1.57	30.2	1.26	24.2
2008	3	31.91	61.5	16.98	32.7	12.10	23.3	2.84	54.5	1.58	30.2	1.26	24.3
2008	4	32.12	61.8	17.12	32.9	12.16	23.4	2.85	54.6	1.58	30.3	1.27	24.3
2009	1	32.32	62.1	17.26	33.1	12.23	23.5	2.86	54.7	1.59	30.4	1.27	24.4
2009	2	32.53	62.4	17.40	33.3	12.29	23.6	2.86	54.8	1.59	30.4	1.28	24.4
2009	3	32.74	62.6	17.54	33.6	12.36	23.6	2.87	54.9	1.60	30.5	1.28	24.4
2009	4	32.96	62.9	17.69	33.8	12.43	23.7	2.88	55.0	1.60	30.6	1.28	24.5
2010	1	33.18	63.2	17.83	34.0	12.49	23.8	2.89	55.1	1.61	30.6	1.29	24.5
2010	2	33.40	63.5	17.98	34.2	12.56	23.9	2.90	55.2	1.61	30.7	1.29	24.5
2010	3	33.62	63.8	18.13	34.4	12.63	24.0	2.91	55.3	1.62	30.7	1.29	24.6
2010	4	33.85	64.1	18.29	34.6	12.70	24.0	2.92	55.4	1.63	30.8	1.30	24.6
2011	1	34.08	64.4	18.44	34.8	12.77	24.1	2.93	55.5	1.63	30.9	1.30	24.6
2011	2	34.31	64.6	18.60	35.0	12.85	24.2	2.94	55.6	1.64	30.9	1.31	24.7
2011	3	34.53	64.9	18.75	35.3	12.91	24.3	2.95	55.7	1.64	31.0	1.31	24.7
2011	4	34.75	65.2	18.90	35.5	12.98	24.4	2.96	55.8	1.65	31.1	1.31	24.8
2012	1	34.97	65.5	19.05	35.7	13.05	24.5	2.97	55.9	1.65	31.1	1.32	24.8
2012	2												
2012	3												
2012	4												
2013	1												
2013	2	36.00	66.9	19.78	36.7	13.38	24.9	3.00	56.2	1.67	31.4	1.32	24.9
2013	3	36.16	67.0	19.90	36.9	13.45	24.9	3.00	56.2	1.67	31.4	1.32	24.8
2013	4	36.31	67.2	20.02	37.0	13.52	25.0	3.00	56.2	1.68	31.4	1.32	24.7
2014	1	36.47	67.3	20.14	37.2	13.58	25.1	3.00	56.1	1.68	31.5	1.31	24.6
2014	2	36.63	67.5	20.27	37.3	13.65	25.2	3.00	56.1	1.68	31.5	1.31	24.5
2014	3	36.79	67.6	20.39	37.5	13.72	25.2	3.00	56.0	1.69	31.5	1.31	24.4
2014	4	36.95	67.8	20.52	37.6	13.79	25.3	3.00	56.0	1.69	31.5	1.30	24.3
2015	1	37.11	67.9	20.64	37.8	13.86	25.4	3.00	56.0	1.69	31.5	1.30	24.2
2015	2	37.28	68.1	20.77	37.9	13.93	25.5	3.00	55.9	1.69	31.6	1.30	24.1
2015	3	37.44	68.2	20.90	38.1	14.00	25.5	3.00	55.9	1.70	31.6	1.29	24.1
2015	4	37.60	68.4	21.02	38.2	14.07	25.6	3.00	55.9	1.70	31.6	1.29	24.0

Modelled counts and trends after adjusting for seasonality and autocorrelation.

Trends in Scotland were flatter during the baseline. Total hospitalisations increased by 0.2% per quarter (from 54.0 to 55.9 per 1,000), elective admissions increased by 0.20% per quarter (from 29.9 to 31.1 per 1,000), and emergency admissions increased by 0.2% per quarter (from 24.1 to 24.8 per 1,000). Again, there was no evidence of any change in these trends after the HSCA: slope changes were −0.3% (95% CI −0.7%–0.2%), −0.1% (95% CI −0.7%–0.4%) and −0.5% (95% CI −1.1%–0.1%), respectively.

The results of the CITS analysis were again similar. The differential slope changes in England (that is, the additional quarterly change following the HSCA after controlling for trends in Scotland) were: 0.0% (95% CI −0.6%–0.6%) per quarter for total hospitalisations, −0.1% (95% CI −0.7%–0.5%) per quarter for elective hospitalisations, and 0.2% (95% CI −0.5%–1.0%) per quarter for emergency hospitalisations.

Results using Wales as a control instead of Scotland were similar (S2 Table and S2 Fig).

## Discussion

To our knowledge, this is the first study of the potential impact on secondary care activity of a universal, national policy that gave control of an unprecedented two-thirds of the English NHS budget to GP-led CCGs. Contrary to the underlying hypothesis, we found no evidence of a reduction in hospitalisations or specialist visits in England following the HSCA. Moreover, we found evidence of an increase over and above the underlying trend in specialist visits in England, with no comparable increase in Scotland, where this policy did not occur. This increase was equivalent to approximately 3.7 million additional specialist visits since the policy was implemented (compared to those expected), of which the majority (approximately 2.9 million) were GP referred.

We used a robust CITS design. By modelling long-term underlying trends, we controlled for secular changes in practice and artefactual changes due to regression to the mean. Selection bias is only an issue in the unlikely event that the population changed suddenly and substantially in contrast to the underlying trend and differentially from trends in the control. S1 Table shows that population characteristics maintained stable trends over the study period, suggesting that this is not an alternative explanation for our findings. Furthermore, we controlled for unknown confounding events coincident with the policy by including Scotland as a comparator. Our study is also based on a very large population with stable trends in the outcomes before and after the intervention; therefore, we are well powered to detect effects. Finally, the compulsory nature and large scale of the intervention again limits selection bias and increases both the internal and external validity of our results.

Our study has several limitations. First, it is possible that the observed changes in trends could have been due to other concurrent policies targeting these outcomes but that did not occur in the control population. Following a literature review, we found one such national policy: an “enhanced service” encouraging GPs to provide extra support for patients deemed at risk of unplanned admission to the hospital; however, this was introduced a year after the Act and only targeted 1 of our outcomes (emergency admissions) in which we did not see a change [21]. We also considered the fact that the Act included a broad range of changes alongside GP-led commissioning and that observed changes in trends might be due to other aspects of the reforms. However, most of the other changes were support structures for the changes to commissioning (such as accountability systems and services regulating specialist care providers) that would be considered integral to the intervention itself, or structural changes to public health and preventative services that are likely to have little direct impact on hospitalisations or specialist visits [2, 4]. Second, smaller-scale effects on certain specialties or diagnoses may have been diluted by the scale of our data. However, as the first study of this nationwide policy, and given the government’s aim to address rising demands and treatment costs within the NHS as a whole [4], our goal was to examine the association between the policy and trends in specialist visits and hospitalisations. Third, while we have nearly 3 years of postintervention data, it is possible that some effects of GP-led commissioning have not yet become evident. For example, GPs may have chosen to invest more in preventative services, which can take several years to result in population-level reductions in disease. Finally, our study uses routine data that were not specifically created to answer this research question. However, we use the data in high-level aggregate analysis and only use final, rather than provisional, data, which are regarded as complete. Therefore, quarterly changes are unlikely to be due to issues such as data completeness or misclassification [22].

Following the introduction of the HSCA, the Department of Health (DH) called for research to evaluate its impact [23]. Nevertheless, initial proposals were rejected, and, while the DH has published an evaluation focussing on the processes of the reforms, we were unable to find any studies looking at the impact of this policy on hospital activity [23, 24]. There have been studies of previous policies that handed greater budgetary responsibility to GPs in the UK and in Israel [25–32]. However, the results of these studies are mixed and difficult to interpret, as all used simple pre-post designs, which do not take into account underlying trends in hospitalisations or specialist visits, and they examined smaller policies, which were voluntary and subject to volunteer selection bias. The lack of control for underlying trends in these studies is particularly important because study groups often appear to have had unusually high referral rates prior to the intervention (partly because budget allocations based on existing referral rates incentivized practices to inflate referrals before becoming budget holders) [27]. Any reduction could therefore have simply been due to regression to the mean.

Our findings suggest that, on a national scale, the concerns raised around restrictions in access to specialist services and rationing of care have not been realised. However, the lack of decrease in hospitalisations and the unanticipated increase in specialist visits also suggest the theorised shift in care away from hospitals to less expensive community settings does not appear to have occurred and, if anything, the increase in specialist visits may have led to cost increases. There are a number of possible reasons why specialist visits and hospitalisations did not decrease. First, while CCGs intended to increase clinical involvement in commissioning, survey evidence suggests that some GPs do not feel fully engaged with their CCG [33]. For example, the majority of GPs are CCG members but do not have a formal role in the governing body, and this group reported much lower levels of influence and ownership than governing body members. A lack of engagement with members may mean that many GPs feel detached from their CCG and under little pressure to make cost savings or unable to influence the way local health services are managed [33]. Second, the financial incentive for CCGs to reduce costs and GPs to change referral patterns may have been too small or too indirect, and, while practice income may have increased by shifting some care from hospitals to community-based care provided by GPs, concerns about potential conflicts of interest could have discouraged this [7]. Finally, it is also possible that referrals to specialists were already appropriate prior to the intervention, resulting in little scope for further reduction. This is supported by evidence that variations in referral rates in the NHS are primarily explained by characteristics of the patient population and not factors affecting GP services [34].

The increase in specialist visits in our study was surprising and may be an unintended consequence of the policy. We identified annual data on NHS costs for outpatient specialist visits from an independent source (S3 Fig). This also appears to show an increase in costs, corroborating our findings regarding upward trends in specialist visits. One explanation might be that the new responsibility for managing budgets has inadvertently increased administrative workload for GPs, resulting in less time to see patients. Under such circumstances, GPs may reduce their threshold for referral to avoid missing a diagnosis. There is some existing evidence to suggest that increased workload and reduced consultation time is associated with increased referral rates [12, 13], although other studies have shown no effect [35]. We considered decreasing GP numbers or increasing supply of specialists as other potential explanations for this finding. However, although there was a slight decrease in the number of full-time equivalent GPs (from 0.69 to 0.67 per 1,000 population) between 2009 and 2010, this does not coincide with the increase in specialist visits, and the number of GPs remained stable from 2010 and, in fact, increased back to 0.69 per 1,000 population in 2014 [36]. Number of specialists (full-time equivalent consultants) increased gradually over the study period from 0.67 per 1,000 population in 2009 to 0.76 per 1,000 population in 2014 and there was no deviation in this trend around the introduction of the HSCA [37].

In conclusion, we found no evidence that the introduction of GP-led commissioning in England was associated with a reduction in overall hospitalisations or specialist visits. In fact, there was an increase in specialist visits, which appears to have been paralleled by an increase in expenditure. This study begins to decipher the macro effects of these significant reforms to the organisation of NHS commissioning. However, many questions remain unanswered. Examples include the appropriateness of any change in rates of specialist visits and hospitalisations, the effect of this change on health outcomes, whether changes differed according to CCG and why, and the generalizability of our findings to other health systems. This study alone is unable to determine whether the HSCA can be regarded as a good or bad policy, and further research is needed to evaluate other important outcomes such as costs and quality of care. Nevertheless, in the context of similar findings from other large-scale health policy experiments [38], more effort may be needed to target specific costly or poorly evidenced practices (such as tonsillectomy, tympanostomy, or antibiotics prescribed for viral infections) rather than to count on broad, system-wide policy changes that often have unintended consequences.

## Supporting information

S1 Table Population characteristics of England and Scotland, 2007–2014. (DOCX)

S2 Table Trend changes in specialist visits and hospitalisations following the intervention in England versus Wales. Coefficients for trend change are relative change in the slope gradient following the intervention. Trend change study versus control is the slope change in England over and above any change in Wales accounting for differences in baseline trends. All segmented regression models used log transformed Gaussian distribution.
(DOCX)

S1 Fig Time series of outpatient specialist visits in England and Wales. Red o = England, blue x = Wales. Lines = deseasonalized linear trend. Vertical lines delineate the intervention phase (between quarter 2 [Q2] 2014 and Q2 2013).
(DOCX)

S2 Fig Time series of inpatient hospitalisations in England and Wales. Red o = England, blue x = Wales. Lines = deseasonalized linear trend. Vertical lines delineate the intervention phase (between quarter 2 [Q2] 2014 and Q2 2013).
(DOCX)

S3 Fig National Health Service (NHS) reference costs. (DOCX)

S1 Text Controlled interrupted time series model. (DOCX)

S1 Checklist REporting of studies Conducted using Observational Routinely-collected health Data (RECORD) statement. (DOCX)

S1 Protocol (DOCX)

S1 Data (XLSX)

S2 Data (XLSX)

S3 Data (PDF)

S4 Data (DOC)

## Abbreviations

CCG: Clinical Commissioning Group
CITS: controlled interrupted time series
DH: Department of Health
GP: general practitioner
HES: Hospital Episode Statistics
HSCA: Health and Social Care Act
NHS: National Health Service
PCT: primary care trust
RECORD: REporting of studies Conducted using Observational Routinely-collected health Data
SHA: Strategic Health Authority
SMR: Scottish Medical Records
