# Correction: Association between the 2012 Health and Social Care Act and specialist visits and hospitalisations in England: A controlled interrupted time series analysis

**DOI:** 10.1371/journal.pmed.1002527

**Published:** 2018-02-26

**Authors:** The PLOS Medicine Staff

The fifth author’s name is spelled incorrectly in the online version. The correct name is J. Frank Wharam. The correct citation is: Lopez Bernal JA, Lu CY, Gasparrini A, Cummins S, Wharam JF, Soumerai SB (2017) Association between the 2012 Health and Social Care Act and specialist visits and hospitalisations in England: A controlled interrupted time series analysis. PLoS Med 14(11): e1002427. https://doi.org/10.1371/journal.pmed.1002427. The name and citation appear correctly in the PDF version.
