# Supplementary material for: Association between the 2012 Health and Social Care Act and specialist visits and hospitalisations in England: A controlled interrupted time series analysis
Source: PLoS Med. 2017 Nov 14;14(11):e1002427. doi: 10.1371/journal.pmed.1002427 (PMC5685471; doi:10.1371/journal.pmed.1002427)
Supplement: S1 Table — (DOCX) [file pmed.1002427.s001.docx]

S1 Table: Population characteristics: England and Scotland 2007-2014[1]

## References

1. Office for National Statistics. Population estimates [Available from: <http://www.ons.gov.uk/peoplepopulationandcommunity/populationandmigration/populationestimates>.
