# Supplementary material for: Association between the 2012 Health and Social Care Act and specialist visits and hospitalisations in England: A controlled interrupted time series analysis
Source: PLoS Med. 2017 Nov 14;14(11):e1002427. doi: 10.1371/journal.pmed.1002427 (PMC5685471; doi:10.1371/journal.pmed.1002427)
Supplement: S2 Table — Coefficients for trend change are relative change in the slope gradient following the intervention. Trend change study versus control is the slope change in England over and above any change in Wales accounting for differences in baseline trends. All segmented regression models used log transformed Gaussian distribution. (DOCX) [file pmed.1002427.s002.docx]

S2 Table: Trend changes in specialist visits and hospitalisations following the intervention England vs Wales

|  |  | Trend change England | | |  | Trend change Wales | | |  | Trend change England v Wales | | |
| --- | --- | --- | --- | --- | --- | --- | --- | --- | --- | --- | --- | --- |
|  |  | Effect | 95% CI | p-value |  | Effect | 95% CI | p-value |  | Effect | 95% CI | p-value |
| Outpatient specialist visits | | |  |  |  |  |  |  |  |  |  |  |
|  | Total | 1.011 | [1.007,1.015] | <0.001 |  | 0.9879 | [0.980,0.996] | 0.003 |  | 1.0233 | [1.014,1.033] | <0.001 |
|  | GP referred | 1.016 | [1.012,1.020] | <0.001 |  | 0.9908 | [0.977,1.004] | 0.186 |  | 1.0254 | [1.011,1.040] | 0.001 |
| Inpatient hospitalizations | | |  |  |  |  |  |  |  |  |  |  |
|  | Total | 0.9982 | [0.995,1.002] | 0.332 |  | 0.9988 | [0.995,1.003] | 0.565 |  | 1.000 | [0.994,1.005] | 0.846 |
|  | Elective | 0.9977 | [0.994,1.001] | 0.212 |  | 0.9983 | [0.991,1.006] | 0.672 |  | 0.999 | [0.991,1.007] | 0.808 |
|  | Emergency | 1.000 | [0.995,1.004] | 0.878 |  | 0.9987 | [0.997,1.001] | 0.182 |  | 1.000 | [0.996,1.005] | 0.884 |

Coefficients for trend change are relative change in the slope gradient following the intervention. Trend change study v control is the slope change in England over and above any change in Wales accounting for differences in baseline trends. All segmented regression models used log transformed Gaussian distribution.
