# Supplementary material for: Association between the 2012 Health and Social Care Act and specialist visits and hospitalisations in England: A controlled interrupted time series analysis
Source: PLoS Med. 2017 Nov 14;14(11):e1002427. doi: 10.1371/journal.pmed.1002427 (PMC5685471; doi:10.1371/journal.pmed.1002427)
Supplement: S2 Fig — Red o = England, blue x = Wales. Lines = deseasonalized linear trend. Vertical lines delineate the intervention phase (between quarter 2 [Q2] 2014 and Q2 2013). (DOCX) [file pmed.1002427.s004.docx]

S2 Figure: Time series of inpatient hospitalisations in England and Wales

Total

England

Wales

Elective

England

Wales

Emergency

England

Wales

Red o = England, blue x = Wales. Lines = deseasonalized linear trend. Vertical lines delineate the intervention phase (between Q2 2014 and Q2 2013)
