# Supplementary material for: Association between the 2012 Health and Social Care Act and specialist visits and hospitalisations in England: A controlled interrupted time series analysis
Source: PLoS Med. 2017 Nov 14;14(11):e1002427. doi: 10.1371/journal.pmed.1002427 (PMC5685471; doi:10.1371/journal.pmed.1002427)
Supplement: S3 Fig — (DOCX) [file pmed.1002427.s005.docx]

S3 Figure: NHS reference costs

1. Reference costs for outpatient specialist visits(1)
2. Reference costs for inpatient admissions (1)

1. Department of Health. Reference costs 2014-15 2015 [Available from: <https://www.gov.uk/government/uploads/system/uploads/attachment_data/file/477919/2014-15_Reference_costs_publication.pdf>.
