# Supplementary material for: Association between the 2012 Health and Social Care Act and specialist visits and hospitalisations in England: A controlled interrupted time series analysis
Source: PLoS Med. 2017 Nov 14;14(11):e1002427. doi: 10.1371/journal.pmed.1002427 (PMC5685471; doi:10.1371/journal.pmed.1002427)
Supplement: S1 Text — (DOCX) [file pmed.1002427.s006.docx]

S1 Text: Controlled interrupted time series model


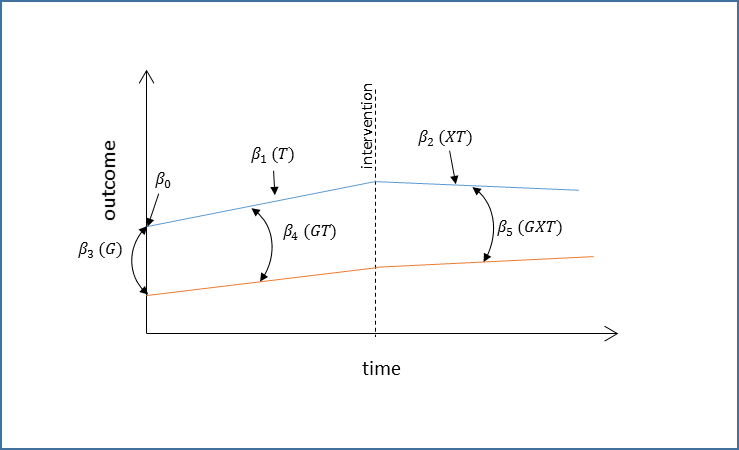


Intervention group in blue, control group in red. T = time since the start of the study, X = intervention (pre-intervention period = 0, post-intervention period = 1), G = group (control group = 0, intervention group = 1). β_0_ and β_3_ relate to intercepts, β_1-2_ and β_4-5_ relate to slopes. Curved arrows represent differences between the intervention group and control group. (Adapted from Linden and Adams 2011)(1)

Segmented regression equation for slope change with a control series:

$$Y_{t}=\beta_{0}+\beta_{1}T+ \beta_{2}TX_{t}+\beta_{3}G+\beta_{4}GT+ \beta_{5}GX_{t}T$$

$Y_{t}$ is the outcome variable at time $t$, $T$ is a variable representing the time since the start of the study and $X$ is a dummy variable indicating the pre- ($X$ = 0) or post-intervention period ($X$ = 1). $G$ represents the intervention group ($G=1$) or control group ($G=0$). $\beta_{0}$represents the intercept at $T$=0, $\beta_{1}$ is the underlying pre-intervention trend (slope), $\beta_{2}$is the slope change following the intervention, $\beta_{3}$ represents the difference in intercept between the two groups at $T$=0, $\beta_{4}$ represents the slope difference between the intervention and control group in the pre-intervention period, $\beta_{5}$ represents the difference between the change in slope in the control and intervention group associated with the intervention. Therefore $\beta_{5}$ is the parameter of interest for the measure of effect.

# References

1. Linden A, Adams JL. Applying a propensity score-based weighting model to interrupted time series data: improving causal inference in programme evaluation. J Eval Clin Pract. 2011;17(6):1231-8.
