# Supplementary material for: Association between the 2012 Health and Social Care Act and specialist visits and hospitalisations in England: A controlled interrupted time series analysis
Source: PLoS Med. 2017 Nov 14;14(11):e1002427. doi: 10.1371/journal.pmed.1002427 (PMC5685471; doi:10.1371/journal.pmed.1002427)
Supplement: S1 Protocol — (DOCX) [file pmed.1002427.s008.docx]

# Evaluation of the impact of GP led commissioning on secondary care activity

## Background:

Clinical Commissioning Groups (CCGs) were created following the Health and Social Care Act 2012 (HSCA),[^1^](#_ENREF_1) and replaced Primary Care Trusts on 1 April 2013 as the main budget holders and commissioners of NHS services in England. CCGs are led by a board, primarily made up of General Practitioners (GPs), and represent all GP practices in the local area. Each is responsible for a population of 100,000-900,000. The theory behind GP led commissioning is, firstly, that GPs understand their patients’ needs best so are best placed to commission specialist services on their behalf, and secondly, that by holding the budgets and being given the freedom to reinvest any savings they are incentivized to ration spending. It has been hypothesized that, given the incentive to minimize costs, GP budget holding may lead to a shift away from expensive secondary care activity towards a more community based approach.[^2-4^](#_ENREF_2) GP commissioning may also influence patient experience of secondary care services as it could incentivize hospitals to reduce waiting times and work to improve patient satisfaction given the threat that commissioners may switch contract to providers that provide a better patient experience.[^4^](#_ENREF_4) Nevertheless, unintended consequences are also possible, for example, there may be inappropriate reductions in care that should have been in hospital and patient satisfaction could suffer if GPs, as budget holders, prioritize cost over patient experience.

## Aim:

To evaluate the impact of GP led commissioning on secondary care activity

## Questions

1. Has the introduction of GP led commissioning been associated with a change in rates of hospital admissions?
2. Has the introduction of GP led commissioning been associated with a change in patients’ experience of secondary care in the NHS?

## Outcomes

##### Primary outcomes:

Secondary care activity, including:

- GP referrals made
- GP referrals seen
- Total admissions
- elective admissions
- outpatient appointments
- emergency admissions

##### Secondary outcomes:

Patient experience, including

- waiting times
- patient satisfaction

## Analysis

An interrupted time series (ITS) design with segmented regression analysis will be used to evaluate the effect of GP led commissioning on secondary care activity. The intervention was fully implemented in April 2013, yet there was a phase in period during which many CCGs were present in shadow form prior to April 2013 and after full implementation changes to commissioning are likely to only have manifested gradually (existing contracts may have taken some time to expire and CCGs may have chosen to simply renew existing contracts in the early stages before they became well established). This “intervention phase” and gradual change should therefore be reflected in the chosen impact model. Possible impact models are outlined in Figure 1, ultimately one of these will be chosen a priori based on further consideration of existing knowledge of the intervention and/or secondary data, other impact models will be used in a sensitivity analysis.

The greatest limitation of the ITS design is the potential for confounding by events concurrent to the intervention. In order to strengthen the validity of the design we will incorporate control series to exclude effects from possible confounding events. Geographical control groups will include secondary care activity in Scotland, Wales and Northern Ireland (where the HSCA was not implemented). Control outcomes such as critical care activity (a service that would be unlikely to be substituted by changes to community care) will also be included in the analyses.

Stratified analyses by different types of secondary care activity (elective, emergency, outpatient) and by specialty will be undertaken in order to establish the nature of any effect.


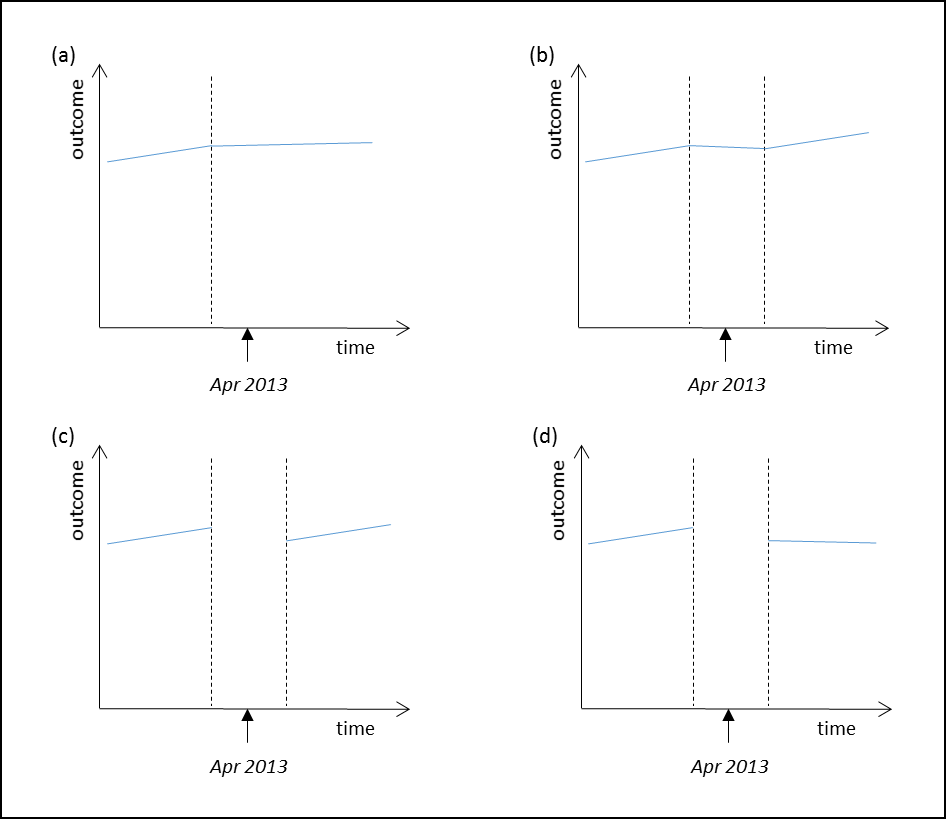


Figure 1: possible impact models

1. gradual slope change beginning during the phase in of the intervention
2. gradual slope change during the “intervention period” followed by a step change
3. step change with exclusion of the “intervention period”
4. step and slope change with exclusion of the “intervention period”

## Data

Monthly data will be needed for each of the following variables – ideally from 2007-2015

| Variable | Data source | Range |
| --- | --- | --- |
| **Outcomes** |  |  |
| Elective admissions (England) | NHS England (HES)  HSCIC | Apr08-Aug15  Apr07-Jul15 |
| Outpatient appointments (England) | NHS England (HES)  HSCIC | Apr08-Aug15  Apr07-Jul15 |
| Emergency admissions (England) | NHS England (HES)  HSCIC | Apr08-Aug15  Apr07-Jul15 |
| GP referrals made (England) | NHS England (HES) | Apr08-Aug15 |
| GP referrals seen (England) | NHS England (HES) | Apr08-Aug15 |
|  |  |  |
| **Denominator data** |  |  |
| Population (England) | ONS | 07-14 |
|  |  |  |
| **Stratification variables** |  |  |
| Gender |  |  |
| Age-group |  |  |
| Diagnosis |  |  |
| Specialty |  |  |
|  |  |  |
| **Controls** |  |  |
| Elective admissions (Scotland) | ISD (NHS Scotland) | 04-Jun15 |
| Outpatient appointments (Scotland) | ISD (NHS Scotland) | 04-Jun15 |
| Emergency admissions (Scotland) | ISD (NHS Scotland) | 04-14 |
| GP referrals made (Scotland) |  |  |
| GP referrals seen (Scotland) |  |  |
| Population (Scotland) | ONS | 07-14 |
| Elective admissions (Wales) |  | 99-14 |
| Outpatient appointments (Wales) | Statswales | 89-12 |
| Emergency admissions (Wales) |  | 99-14 |
| GP referrals made (Wales) | Statswales | Jan05-Sep15 |
| GP referrals seen (Wales) |  |  |
| Population (Wales) | ONS | 07-14 |
| Elective admissions (NI) | dhsspsni | 05-15 |
| Outpatient appointments (NI) | dhsspsni | 09-15 |
| Emergency admissions (NI) | dhsspsni | 05-15 |
| GP referrals made (NI) |  |  |
| GP referrals seen (NI) |  |  |
| Population (NI) | ONS | 07-14 |
| Critical care admissions (England) | HSCIC | Apr08-Mar14 |

| **Secondary outcomes** |  |
| --- | --- |
| Waiting times (England + controls) |  |
| Patient satisfaction score (England + controls) |  |
|  |  |

## Data sources

##### Secondary care activity:

Data on each inpatient admission, outpatient appointment and accident and emergency attendance in NHS hospitals in England are collected as Hospital Episode Statistics (HES) published by the Health and Social Care Information Centre (HSCIC, <http://www.hscic.gov.uk/hes>). Overall activity in each of these categories is made publically available on a monthly basis with a four month delay via the health and social care information centre website and dates back to April 2007 (Figure 1). More detailed annual data is also available stratified by various covariates including diagnosis, specialty and hospital provider. Adult Critical care data is also published on an annual basis. In addition to the data which is made publically available HSCIC operate a Data Access Request Service on a cost recovery basis whereby data can be requested for additional covariates (such as age, gender and index of multiple deprivation) and in greater detail – such as monthly time series of stratified data and even individual patient data. Such requests take between 14 to 60 days to gain access to the data.

Figure 1: Admitted patients finished consultant episodes in England by month 2007-2014

[More data now available via NHS England, including monthly data stratified by other covariates: <http://www.england.nhs.uk/statistics/statistical-work-areas/hospital-activity/monthly-hospital-activity/mar-data/>]

##### Patient experience:

Monthly waiting time data is published by NHS England including:

- Referral to Treatment Waiting Times (<http://www.england.nhs.uk/statistics/statistical-work-areas/rtt-waiting-times/>)
- Cancer waiting times (<http://www.england.nhs.uk/statistics/statistical-work-areas/cancer-waiting-times/>)
- Diagnostic waiting times (<http://www.england.nhs.uk/statistics/statistical-work-areas/diagnostics-waiting-times-and-activity/monthly-diagnostics-waiting-times-and-activity/>)

The NHS Patient Survey Programme coduct annual surveys of patient experience including the Inpatient, Outpatient, Community Mental Health and Accident & Emergency surveys. (<http://www.england.nhs.uk/statistics/statistical-work-areas/pat-exp/> and <https://www.gov.uk/government/collections/patient-experience-statistics>)

Patient Reported Outcome Measures (PROMs) assess the quality of care delivered to NHS patients from the patient perspective. Currently covering four clinical procedures (hip replacements, knee replacements, groin hernia and varicose veins), PROMs calculate the health gains after surgical treatment using pre- and post-operative surveys.

##### Control data:

In Scotland quarterly hospital activity data is published online by the NHS Information Services Devision (ISD, <http://www.isdscotland.org/Health-Topics/Hospital-Care/>) with one quarter delay. Welsh hospital activity data in Wales is collected monthly and collated in the Patient Episode Database for Wales (PEDW) and annual data is published at the end of each year dating back to 1991 by the NHS Wales Informatics Service ([http://www.infoandstats.wales.nhs.uk/page.cfm?orgid=869& pid=40977](http://www.infoandstats.wales.nhs.uk/page.cfm?orgid=869&%20pid=40977)). In Northern Ireland annual hospital activity data is published by the Department of Health, Social Services and Public Safety Information Office dating back to 2005 (<http://www.dhsspsni.gov.uk/index/statistics/hospital/hospital-activity.htm>).

## Threats and potential solutions

| Threat | Solution |
| --- | --- |
| Some CCGs were present in shadow form for several months prior to becoming the official commissioning body so may have influenced commissioning in the months leading up to the intervention. | Consider modelling the intervention earlier |
| Changes to commissioning could have taken some time to manifest: existing contracts may have taken some time to expire and CCGs may have chosen to simply renew existing contracts in the early stages before they became well established | Consider modelling a slope change during the changeover period to allow for a gradual change whilst contracts expired then a step change once contracts should have all expired. Alternatively could exclude the intervention period from the model |
| The HSCA resulted in other changes to the structure of the NHS and public health services (see appendix). Therefore it may be difficult to disentangle whether some effects are due to the introduction of CCGs or due to other changes | It is unlikely that the other changes would have had an impact on secondary care referrals so this should not be an issue for the primary outcome. Some of the other changes such as a purported “greater voice for patients” could result in changes to secondary outcomes relating to patient experience, nevertheless this change was more of a gradual progression rather than a sudden new change with the implementation of the act.  Using a control outcome such as critical care activity, could help to exclude some of the other changes that could have affected all types of care.  Another option is to treat the intervention as the HSCA as a whole rather than focus on a part of the act. |

## References

1 Health and Social Care Act. (2012).

2 Dusheiko, M., Gravelle, H., Jacobs, R. & Smith, P. The effect of financial incentives on gatekeeping doctors: Evidence from a natural experiment. *Journal of Health Economics* **25**, 449-478, doi:<http://dx.doi.org/10.1016/j.jhealeco.2005.08.001> (2006).

3 Smith, J. A. & Mays, N. GP led commissioning: time for a cool appraisal. *BMJ* **344**, doi:10.1136/bmj.e980 (2012).

4 Mannion, R. General practitioner-led commissioning in the NHS: progress, prospects and pitfalls. *British Medical Bulletin* **97**, 7-15, doi:10.1093/bmb/ldq042 (2011).

## Appendix

2012 Health and Social Care Act key policy areas:

1. Clinically led commissioning:
   - Abolition of Primary Care Trusts (PCTs) and Strategic Health Authorities (SHAs) + moving commissioning to GP led Clinical Commissioning Groups (CCGs)
2. Provider regulation to support innovative services:
   - Monitor (a national regulatory body) given greater role in promoting competition and role in licensing providers of NHS services (including private sector and charity providers).
   - Emphasis on choice and competition as a driver of improved patient care
3. Greater voice for patients:
   - Building on previous efforts to increase patient involvement in the NHS
   - Included establishment of Healthwatch (as a committee of the Care Quality Commission) to represent patient views in advising NHS organisations
4. New focus for public health:
   - Establishment of Public Health England with a remit of improving the nation’s health at a national level (and abolishment of the Health Protection Agency)
   - Moving public health departments at a local level from PCTs to Local Authorities (establishment of PHE as ne body to drive improvements in PH)
5. Greater accountability locally and nationally:
   - Strengthens and clarifies accountability for and within the NHS
   - CCGs accountable to NHS England and assessed against a commissioning outcomes framework
   - Statutory health and wellbeing boards within local authorities aimed to ‘strengthen the local democratic legitimacy of the NHS’ with responsibility for joining up the commissioning of local NHS services and social care.
6. Streamlined arms-length bodies:
   - Several bodies abolished with key functions transferred to other bodies (including: General Social Care Council, Office of the Healthcare Professions Adjudicator, Alcohol Education and Research Council, National Patient Safety Agency, NHS Institute for Innovation and Improvement, National Information Governance Board and the Appointments Commission)

<http://www.bbc.co.uk/news/health-34421115>

<http://www.pulsetoday.co.uk/news/commissioning/commissioning-topics/referrals/gp-practices-offered-payments-to-cut-urgent-cancer-referrals/20030100.article>

<http://www.lexology.com/library/detail.aspx?g=9c72de3e-1c46-45f5-b158-66d0b4c255c5>
