# Supplementary material for: Association between the 2012 Health and Social Care Act and specialist visits and hospitalisations in England: A controlled interrupted time series analysis
Source: PLoS Med. 2017 Nov 14;14(11):e1002427. doi: 10.1371/journal.pmed.1002427 (PMC5685471; doi:10.1371/journal.pmed.1002427)
Supplement: S3 Data — (PDF) [file pmed.1002427.s011.pdf]

NIC-04929-V2W0L - London School of Hygiene and Tropical Medicine

Inpatient Specification

Tables Used:

HES_APC_0708, HES_APC_0809, HES_APC_0910, HES_APC_1011, HES_APC_1112, HES_APC_1213, HES_APC_1314, HES_APC_1415, *HES_APC_1516 (provisional)*

Fields Used:

EPIEND, ADMIDATE, ADMIMETH, MAINSPEF, FCE, FAE

Filters Applied:

FCE = 1

Table Layout

Table 1

Year	Month	Elective Admissions
=Year(EPIEND)	=Month(EPIEND)	Case when ADMIMETH in (‘11’,’12’,’13’) then sum(FAE)

Table 2

Year	Month	Main Specialty	Episodes	Admissions	Elective Admissions
Year(EPIEND)	Month(EPIEND)	MAINSPEF	Sum(FCE)	Sum(FAE)	Case when ADMIMETH in (‘11’,’12’,’13’) then sum FAE

Outpatient Specification

Tables Used:

HES_OP_0708, HES_OP_0809, HES_OP_0910, HES_OP_1011, HES_OP_1112, HES_OP_1213, HES_OP_1314, HES_OP_1415, *HES_OP_1516 (provisional)*

Fields Used:

APPTDATE, REFSOURC, MAINSPEF, ATTENDKEY_FLAG, ATTENDANCE, ATENTYPE

Filters:

ATTENDKEY_FLAG = 1

<u>Table Layout:</u>

Table 1

Year	Month	GP Referred Appointments	GP Referred Attendances
=Year(APPTDATE)	=Month(APPTDATE)	When REFSOURC in ('03','12') then sum(ATTENDKEY_FLAG)	When RESOURCE in ('03','12') then sum(ATTENDANCE)

Table 2

Year	Month	Main Specialty	Total OP appointments by main specialty	GP referral outpatient appointments by main specialty	OP attendance by main specialty	1st OP attendance by main specialty	GP referred OP attendance by main specialty
=Year(APPTDATE)	=month(APPTDATE)	=MAINSPEF	=sum(ATTENDKEY_FLAG)	When REFSOURCE = ('03','12') then sum(ATTENDKEY_FLAG)	=sum(ATTENDANCE)	When ATENTYPE in ('1','21') then sum(ATTENDANCE)	When REFSOURCE in ('03','12') then sum(ATTENDANCE)

Critical Care Admissions are based on the following:

Tables Used:

HES_CC_0708 to *HES_CC_1516 (provisional)*
HES_APC_0708 to *HES_APC_1516 (provisional)*
(NB. (for analyst) Not all data is available in EG– only a few years are. For previous data you have to get it from FAT_HES on 470 & use SQL)

Fields Used:

HES_CC_YYYY.CCDISDATE
HES_APC_YYYY.EPIKEY

Filters Applied:

HES_CC_YYYY.BESTMATCH = 1
HES_CC_YYYY.SUSRECID = HES_APC_YYYY.SUSRECID

Table Layout:

Table 1:

Year	Month	Count
Year(DISDATE)	Month(DISDATE)	Count(EPIKEY)

NHS Only patients

Tables Used:

Inpatients
HES_APC_0708, HES_APC_0809, HES_APC_0910, HES_APC_1011, HES_APC_1112, HES_APC_1213, HES_APC_1314, HES_APC_1415, *HES_APC_1516 (provisional)*

HES_APC_OTH_0708, HES_APC_OTH_0809, HES_APC_OTH_0910, HES_APC_OTH_1011, HES_APC_OTH_1112, HES_APC_OTH_1213, HES_APC_OTH_1314, HES_APC_OTH_1415, *HES_APC_OTH_1516 (provisional)*

Outpatients
HES_OP_0708, HES_OP_0809, HES_OP_0910, HES_OP_1011, HES_OP_1112, HES_OP_1213, HES_OP_1314, HES_OP_1415, *HES_OP_1516 (provisional)*

Critical Care
HES_CC_0708 to *HES_CC_1516 (provisional),* HES_APC_0708 to *HES_APC_1516 (provisional)*
(NB. (for analyst) Not all data is available in EG – only a few years are. For previous data you have to get it from FAT_HES on 470 & use SQL)

Fields Used:

Inpatients
EPIEND, ADMIDATE, ADMIMETH, FCE, FAE, CATEGORY

Outpatients
APPTDATE, REFSOURC, ATTENDKEY_FLAG, ATTENDANCE, ATENTYPE, ADMINCAT

Critical Care
HES_CC_YYYY.CCDISDATE, HES_APC_YYYY.EPIKEY, CATEGORY

Filters Applied:

Inpatients

FCE = 1, CATEGORY Not in list (‘20’, ‘21’, ‘22’, ‘23’, ‘30’, ‘31’, ‘32’, ‘33’) *(NULL values included as ‘Other maternity related events)*

Outpatients

ATTENDKEY_FLAG = 1, ADMINCAT Not in list (‘02’, ‘03’)

Critical Care

HES_CC_YYYY.BESTMATCH = 1, HES_CC_YYYY.SUSRECID = HES_APC_YYYY.SUSRECID, CATEGORY Not in list (‘20’, ‘21’, ‘22’, ‘23’, ‘30’, ‘31’, ‘32’, ‘33’)

<u>Table Layout</u>

Table 1

Year	Month	Finished Consultant Episode	Finished Admissions Episode	Emergency Admissions	Non-Emergency Admissions	Elective Admissions	Total Outpatient Appointment	GP Referred Appointments	Attended Outpatient Appointment	1st outpatient appointment	GP Referred Outpatient Attendances	Critical Care Admissions
=Year (EPIEND), (APPTDATE), (CCDISDATE)	=Month (EPIEND), (APPTDATE), (CCDISDATE)	Sum(FCE)	SUM(FAE)	Case when ADMIMETH like ‘2%’ then sum (FAE)	Case when ADMIMETH not like ‘2%’ then sum(FAE)	Case when ADMIMETH in (‘11’,’12’,’13’) then sum(FAE)	Sum (ATTENDKEY_FLAG)	When REFSOURCE = (‘03’,’12’) then sum (ATTENDKEY_FLAG)	Sum (ATTENDANCE)	When ATENTYPE in (‘1’,’21’) then sum (ATTENDANCE)	When REFSOURCE in (‘03’,’12’) then sum (ATTENDANCE)	Count (EPIKEY)

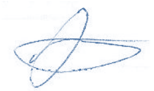

James Lopez Bernal
