# Supplementary material for: Association between the 2012 Health and Social Care Act and specialist visits and hospitalisations in England: A controlled interrupted time series analysis
Source: PLoS Med. 2017 Nov 14;14(11):e1002427. doi: 10.1371/journal.pmed.1002427 (PMC5685471; doi:10.1371/journal.pmed.1002427)
Supplement: S4 Data — (DOC) [file pmed.1002427.s012.doc]

**SCT Information Request Form**

Non-NHS customers should be aware that ISD operates a charging policy for any request requiring new analysis and/or data extraction. Details of this policy can be found at [Charging Policy](http://www.isdscotland.org/About-ISD/Information-Requests/)

Please complete the form below and give as much detail as possible to minimise any delay to your request. If you require assistance completing the form please contact: [NSS.isdsct@nhs.net](mailto:NSS.isdsct@nhs.net)

| Name of customer |  |
| --- | --- |
| Organisation and address |  |
| Email address |  |
| Telephone number |  |
| Is this a repeat request (Yes/No)  If Yes, state previous IR number - | No |
| Data required;   1. total admissions (CIS) 2. emergency admissions (CIS) 3. elective admissions (CIS) 4. total outpatient attendances 5. New GP referred outpatient appointments 6. new outpatient appointments 7. new outpatient attendance 8. New GP referred outpatient attendances   1-3, 5-8. Aggregate monthly data – totals and stratified by specialty (specialty on admission for IP data, all specialties to be included)  4. By quarter, to be taken from publication.  (see also attached spreadsheet)  Please note: If the data required involves small numbers the [ISD Disclosure protocol](http://www.isdscotland.org/About-ISD/Confidentiality/) may be applied. | |
| Time period (calendar year, financial year, quarter, single year, trend data etc)  *e.g. Calendar years 2009, 2010*  Monthly – April 2007 – December 2015 Use date of admission for inpatient data | |
| Geography (NHS Board, Local Authority, Other (please specify).  *e.g. NHS Scotland, Health Board of Treatment, Health Board of Residence.*  All Scotland  Please note: If data is required at lower geography level there is a possibility that the numbers may be small and the [ISD Disclosure protoco](http://www.isdscotland.org/About-ISD/Confidentiality/)l will be applied. | |
| Other (Type of admission, Deprivation, Age group, Gender)  e.g. *by Scottish Index of Multiple Deprivation (SIMD) or Elective/Emergency/Transfer*  Types of admissions/appointments as detailed above  I would like the data for all patients (private and NHS) if available, but also a copy of the totals for all specialties (sheet 1 of the attached Excel spreadsheet) restricted to just NHS patients  If data is to be broken by Age group, please specify which ages are to be included. | |
| Purpose of data  *e.g. Monitor impact of XXX initiative*  Research study to evaluate the impact of the 2012 Health and Social Care Act in England on hospital activity in comparison to hospital activity in Scotland | |
| Do you intend to publish these data?  *e.g. Annual Report*  Yes, following analysis - as an academic paper | |

Please return this form to: [NSS.isdsct@nhs.net](mailto:NSS.isdsct@nhs.net)

When this form is returned and details are agreed, we would normally aim to answer the information request within 20 working days.
